# Visit-to-Visit Blood Pressure Variability as a Risk Factor for All-Cause Mortality, Cardiovascular Mortality, and Major Adverse Cardiovascular Events Among American Indians: the Strong Heart Study

**DOI:** 10.5888/pcd22.240512

**Published:** 2025-06-26

**Authors:** Richard R. Fabsitz, Jessica A. Reese, Jean Leidner, Marilyn G. Klug, Ying Zhang, Astrid M. Suchy-Dicey, Richard B. Devereux, Lyle G. Best, Marc D. Basson

**Affiliations:** 1 Missouri Breaks Industries Research, Inc, Eagle Butte, South Dakota; 2 University of Oklahoma, Oklahoma City; 3 University of North Dakota, Grand Forks; 4 Huntington Medical Research Institutes, Pasadena, California; 5 Weill Cornell Medicine, New York; 6 Northeast Ohio Medical University, Rootstown, Ohio

## Abstract

**Introduction:**

Recent literature suggests blood pressure variability (BPV) is an independent risk factor for cardiovascular disease (CVD). Ours is the first study to assess the prognostic value of the intraindividual SD of systolic blood pressure (SBPSD) and diastolic blood pressure (DBPSD) in American Indians.

**Methods:**

We computed BPV for 3,352 American Indians who had 8 nonurgent visit-to-visit blood pressure checks according to their electronic health records, and linked those measurements with Strong Heart Study cohort data. We used Cox proportional hazards models to determine whether the risk of all-cause mortality, CVD mortality, or major adverse cardiovascular events (MACE), was different for SBPSD and DBPSD quartiles, while controlling for covariates.

**Results:**

Mean participant age was 54.5 years (SD = 17.3), 66% were female, mean SBPSD was 13.47 (SD = 5.71), and mean DBPSD was 8.05 (SD = 3.02). Over the 20-year follow-up, 45.4% died, 14.6% experienced CVD-related mortality, and 20.8% experienced MACE. Compared with the lowest SBPSD quartile (quartile 1), the risk of all-cause mortality was 35% higher for the highest quartile (quartile 4), while controlling for covariates (HR = 1.35; 95% CI, 1.13–1.61). The risk of CVD mortality and MACE was higher for quartile 4 SBPSD compared with quartile 1 (CVD mortality, HR = 1.81, 95% CI, 1.29–2.53; MACE HR = 1.39, 95 % CI, 1.07–1.80). The risk for quartile 4 DBPSD was not significant for these outcomes (all-cause mortality, HR = 1.15, 95% CI, 0.97–1.36; CVD mortality, HR=1.22, 95% CI, 0.91–1.65; MACE, HR = 1.11, 95% CI, 0.87–1.40).

**Conclusion:**

Our study identified SBPSD as a significant risk factor for all-cause mortality, cardiovascular mortality, and MACE, whereas DBPSD in our cohort of American Indian subjects was not a significant risk factor after adjustment for covariates.

**Summary**

**What is already known on the topic?**

Blood pressure variability has been shown in multiple studies to be an independent risk factor for all-cause mortality, cardiovascular disease mortality, and major adverse cardiovascular events.

**What is added by this report?**

Ours is the first study to show the prognostic value of blood pressure variability in American Indians, a population with unique genetics, culture, lifestyle, and risk factors. The study expands the prognostic value of blood pressure variability to that population.

**What are the implications for public health practice?**

As electronic health record systems proliferate, the evidence to support the routine calculation of blood pressure variability offers a value-added proposition to those records and the impetus to advance therapies for blood pressure control.

## Introduction

Blood pressure level is a well-recognized risk factor for cardiovascular disease (CVD) (1). Over the last 2 decades, research and clinical interests in blood pressure have expanded to include blood pressure variability (BPV), here defined as the SD of 8 nonurgent, visit-to-visit blood pressure measurements. Interest in BPV as a risk factor independent of blood pressure level surged after Rothman and colleagues reported it as a risk factor for stroke independent of blood pressure (2). Since then, BPV has also been shown to be a risk factor, independent of blood pressure level, for all-cause mortality (3–13), CVD mortality (4,6,8,11–14), and CVD morbidity (2,4–7,9–12,14–17). In addition, BPV has been linked to cognitive decline (18), peripheral vascular disease (19), chronic kidney disease (5), decline in estimated glomerular filtration rate (eGFR) (15), type 2 diabetes (20), worsened cardiac structure and function (21), and progression of coronary artery calcification (22). Although BPV can be measured in several ways (23), the SD of visit-to-visit systolic blood pressures is the most common (11) and reproducible (24) measure.

Because BPV may be associated with genetic factors (25) and is likely to be influenced by cultural and lifestyle factors such as diet, alcohol and cigarette consumption, and exercise, it is important to investigate BPV in different populations. To date, none of the published investigations of BPV has focused on American Indians or had sufficient sample size to provide results specific to this population.

Our study is the first known effort to evaluate the prognostic value of BPV for all-cause and cardiovascular mortality and major adverse cardiovascular events (MACE) in a large, geographically diverse group of American Indians. We merged standardized data from cohort examinations with unstandardized blood pressure data collected in the “real world” from nonurgent clinic visits within a common medical care system to evaluate the prognostic value of clinical BPV for adjudicated mortality and morbidity endpoints over a 20-year follow-up.

## Methods

### Available data

The Strong Heart Study (SHS) is a longitudinal, observational study of CVD and its risk factors (26). It originally sampled 4,549 men and women aged 45 to 74 years from the general population of American Indians in Arizona, Oklahoma, and the Dakotas (27). The first examination was conducted in 1989–1992. The group was re-examined a second time in 1993–1995, and a third time in 1997–1999. Subsequently, the focus of the study changed, and we sampled large, 3-generation families (n = 3,665) with a first examination in 2001–2003. That examination was called the Strong Heart Family Study (SHFS). Its cohort was re-examined in 2006–2009. To incorporate longer follow-up, 825 overlapping individuals between the 2 cohorts were allocated to the SHS cohort. All tribal members aged 45 to 74 years were invited to be examined for the original study. For the SHFS, 120 families of 30 members or more aged 15 years or older were invited to participate. The study was approved by all relevant institutional review boards from the Indian Health Service (IHS), the various research institutions, and participating tribes at the initiation of each phase of the study. All participants provided written informed consent and access to their patient records.

For a subgroup of participants in the SHS and SHFS, we linked medical records for inpatient and outpatient visits to IHS facilities to gain access to routine blood pressure measurements collected as early as 1998. We obtained data from the National Data Warehouse (NDW) of the IHS, which houses the National Patient Information Reporting System (28). NDW requested that all service units (clinical care facilities) send data from all patient encounters dating back to October 1, 2000. Many, but not all, service units sent data as they were able. We requested NDW data for all SHS and SHFS participants. NDW provided data for only those identification numbers resulting in a match to available blood pressure records.

Extracted IHS NDW data available for each outpatient visit included systolic blood pressure (SBP), diastolic blood pressure (DBP), date of blood pressure measurement, and the clinic where blood pressure was measured. Blood pressures collected as part of hospitalizations, emergency department visits, urgent care visits, ambulance trips, or pregnancy clinical visits were excluded from the calculation to avoid measures taken at stressful times. Although blood pressure variability stabilizes at 6 blood pressure measurements (7), the investigators decided to use 8 measurements for added confidence in the data. Data were included for all SHS and SHFS cohort members with blood pressures recorded for at least 8 clinic visits during the first 5 years of available NDW records.

To maintain data integrity and consistency, analyses used the first measurement per day in each encounter (clinic visit) if there were multiple measures within the clinic visit on the same day, although most encounters provided a single measurement per visit. The first 8 eligible visits were used to calculate the standard deviation of the SBP (SBPSD) and DBP (DBPSD). For analysis, we divided SBPSD and DBPSD into quartiles. Blood pressures from clinic visits did not follow a strict standardized protocol and may be considered real world blood pressure as measured in routine visits across various clinics.

### Covariates

We obtained covariate information from the SHS or SHFS examination that was closest to the time the blood pressures were measured. Generally, baseline cohort exams for this analysis were the third examination in the SHS (1997–1999) and the first examination in the SHFS (2001–2003). Potential covariates were drawn from personal interview, medical history, physical examination, and laboratory measurements at the closest examination. Potential covariates for this analysis were limited to those collected at the third examination of the original cohort and the first examination of the family cohort. Time-dependent covariates could not be obtained because the baseline examination for the original cohort was their last study examination, and the family cohort had only 1 additional study examination approximately 5 years later. Potential covariates for this analysis were age, sex, center, history of cardiovascular disease (myocardial infarction or stroke), diabetes, hypertension, kidney disease, SBP and DBP measured during the examination, ankle/brachial index (ABI), body mass index (weight in kilograms divided by height square meters) (BMI), low density lipoprotein (LDL), high density lipoprotein (HDL), triglycerides, current cigarette smoking, and current alcohol consumption. In addition, all statistical models included an indicator variable to account for cohort differences in disease and death rates.

Details of study design and methods are provided elsewhere for the SHS cohort (26) and the SHFS cohort (27). Interviews and physical examinations were conducted by trained personnel following strict protocols after informed consent was provided. For examination components included in both cohorts, protocols were maintained between SHS and SHFS. Prevalent morbidity was based on a positive response in the medical history interview.

Patients’ SHS examination blood pressures were measured in the right arm after a 5-minute rest in a quiet room by using an appropriate-sized cuff and a mercury sphygmomanometer. Blood pressure was measured 3 times, and the average of the last 2 measurements was used for analysis.

Fasting blood samples from a 12-hour fast were obtained during the physical examination for laboratory measures. All variables were assayed at MedStar Research Institute, Washington, DC, and the University of Vermont by using standard laboratory methods as described previously (26,29).

Participants were considered hypertensive if they were taking antihypertension medication or if they had a systolic blood pressure greater than 130 mm Hg or a diastolic blood pressure greater than 80 mm Hg (1). Urinary albumin excretion was estimated by the ratio of albumin (mg) to creatinine (g). Microalbuminuria was defined as a ratio of urinary albumin (mg/mL) to creatinine (g/mL) of 30 to 299 mg/g and macroalbuminuria as a ratio at or above 300 mg/g. Estimated glomerular filtration rate (eGFR) was calculated by using the Modified Diet and Renal Disease equation (30). Participants reporting a history of end-stage renal disease, those found to have microalbuminuria or macroalbuminuria, and those with eGFR of less than 60 mL per minute per 1.73m^2^ were combined into a category of kidney disease.

### Endpoints

We abstracted medical records for all participants for review for relevant endpoints by trained medical abstractors at each center each year since enrollment in the study. Two physicians reviewed endpoints, and a third reviewer adjudicated differences between reviewers as needed (26). The following 3 endpoints contributed to this analysis: all-cause mortality, CVD mortality, and MACE that included cardiovascular death, nonfatal myocardial infarction, and nonfatal stroke.

Annual endpoint surveillance for SHS cohort participants began following the initial examination in 1988–1989 and ended with the most recently released morbidity and mortality file ending December 31, 2021. Endpoint surveillance for the SHFS cohort began with the initial examination in 2001–2003 and ended December 31, 2021. Surveillance included an annual telephone call to determine vital status and recent hospitalizations, and an annual review and abstraction of medical records for potential endpoints. Follow-up for events for this analysis began after the baseline examination or the eighth blood pressure measurement, whichever came later. Thus, the baseline examination for this analysis was the third examination for the SHS (1997–1999) and the first examination for the SHFS (2001–2003). Follow-up for events extended approximately 20 years.

### Statistical analysis

We used SAS (SAS Institute Inc) to conduct all analyses. Comparisons of baseline covariates for study participants between those included and excluded from analysis were done with independent sample *t* tests for normally distributed variables, Wilcoxon signed rank sum for skewed variables, and χ^2^ for categorical variables. We created side-by-side box plots to present the distribution of SBPSD and DBPSD quartiles. We generated Kaplan–Meier survival curves with time to death as the outcome and used log rank tests to determine whether there were differences in time to death between the SBPSD and DBPSD quartiles. We conducted univariate and multivariate analyses of covariates and BPV by using shared frailty Cox proportional hazards models accounting for the correlation among family members. The models met the assumption of proportional hazards. Covariates were selected for adjustment in models based on literature review. Analyses were done in 2 steps of covariate adjustment after univariate analyses: first (Model 1), adjusting for cohort, center, age, sex, and SBP/DBP as appropriate, and second (Model 2), also adjusting for the remaining CVD covariates (hypertension treatment, diabetes, BMI, current smoking, current drinking, LDL, HDL, triglycerides, kidney disease, ankle/brachial index, prevalent myocardial infarction and stroke [not for all-cause mortality], and interaction of prevalent systolic blood pressure or diastolic blood pressure and hypertension treatment). To separate the therapeutic effect from the severity effect of blood pressure medications, one 2-way interaction term (blood pressure treatment × blood pressure level) was included in Model 2. Statistical tests of model components were assessed as significant at the *P* < .05 level. A sensitivity analysis was conducted to investigate whether the time interval to collect 8 blood pressure measurements was related to the results by dichotomizing the time interval to collect the 8 measures, breaking the 5-year maximum at 1 year.

## Results

**Figure 1:**
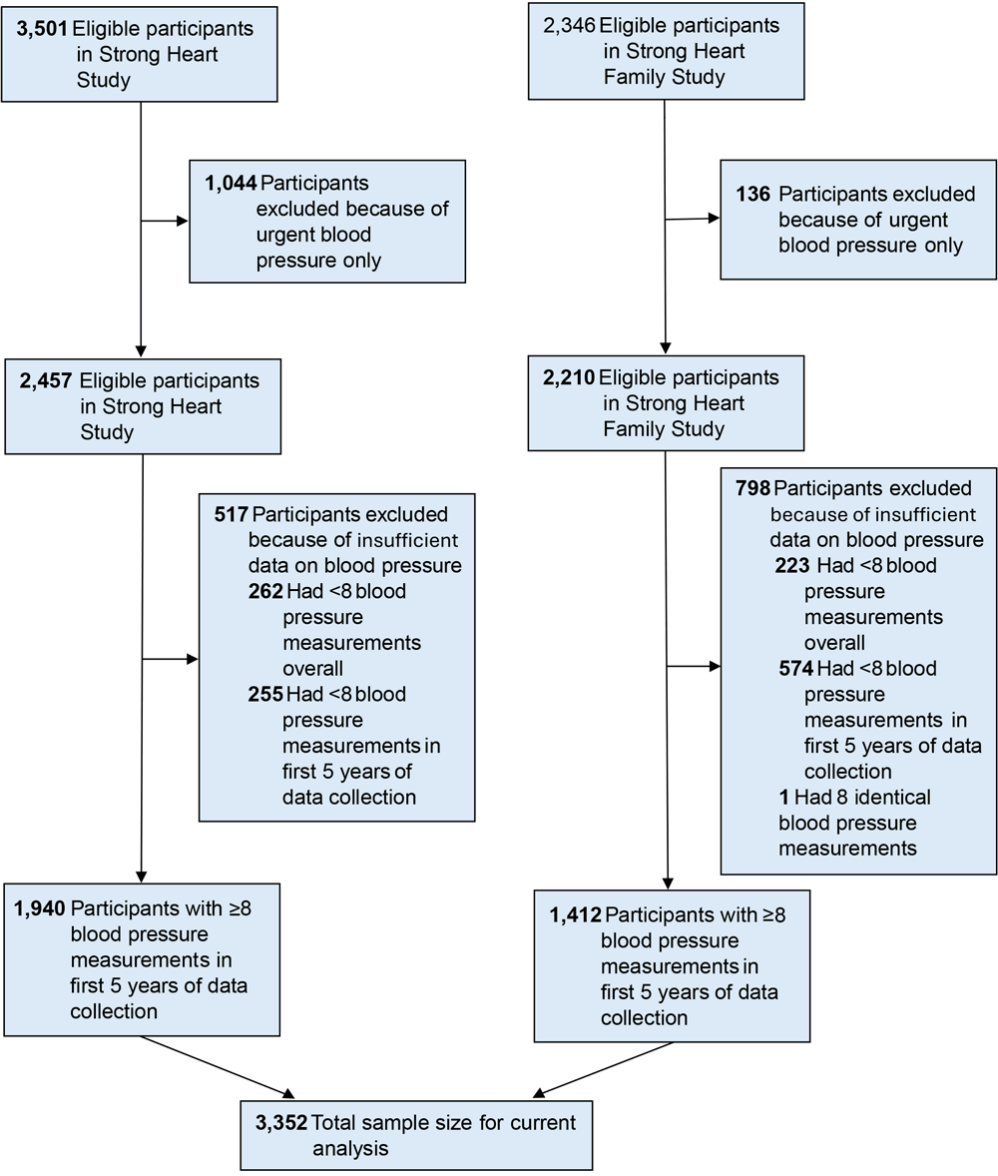
CONSORT Diagram for participants with 8 nonurgent blood pressure measurements in the first 5 years of available medical records from the National Data Warehouse of the Indian Health Service. Study participants were drawn from the Strong Heart Study (26) and the Strong Heart Family Study (27) of American Indians residing in Arizona, Oklahoma, North Dakota, and South Dakota (1997–2003).

Of the original and family cohorts, 3,501 participants from SHS and 2,346 from SHFS were eligible for our study (Figure 1). Of these 2 cohorts, 1,940 of the original and 1,412 of the family cohorts, or a total of 3,352, had 8 or more blood pressure measurements within the first 5 years of available data.

Deaths included 1,523 all-cause deaths (45.4%) and 566 (16.9%) cardiovascular deaths among the cohort members during the 20-year follow-up period. In addition, participants experienced 249 nonfatal myocardial infarctions and 172 nonfatal strokes during the follow-up period. After exclusion of those with prior myocardial infarctions or strokes, 697 MACE events were available for analysis.

We compared the demographic, risk factor, and prevalent morbidity measures for those meeting the inclusion criteria versus those excluded for our current analyses (Table 1). Notably, those excluded had a higher SBP level (125.72 vs 123.49, *P* < .001) but were not statistically different for DBP or history of hypertension.

**Table 1:** Demographic Characteristics and Medical History of Eligible Participants by Inclusion Status, Strong Heart Study Examination 3 (1997–1999) and the Strong Heart Family Study Examination 1 (2001–2003)^a^

Inclusion status of eligible participants	Overall (n = 5,847)	Included (n = 3,352)	Excluded (n = 2,495)	*P* value^b^
**Age at enrollment,**				
Mean (SD)	48.39 (14.76)	47.77 (13.77)	49.21 (15.96)	<.001
Range	14.10–93.30	15.00–90.80	14.10-93.30	
**Sex, n (%)**				
Female	3,422 (59.9)	2,226 (66.4)	1,196 (47.9)	**<**.001
Male	2,425 (40.1)	1,126 (33.6)	1,299 (52.1)	
**Strong Heart Study cohort, n (%)**				
Strong Heart Study	3,501 (59.9)	1,940 (57.9)	1,561 (62.6)	<.001
Strong Heart Family Study	2,346 (40.1)	1,412 (42.1)	934 (37.4)	
**Strong Heart Study data collection center, n (%)**				
Arizona	743 (12.7)	474 (14.1)	269 (10.8)	<.001
Oklahoma	2,504 (2.8)	1,267 (37.8)	1,237 (49.6)	
Dakotas	2,600 (44.5)	1,611 (48.1)	989 (39.6)	
**Systolic blood pressure, nearest visit^c^, mm Hg**				
Mean (SD)	124.44 (18.20)	123.49 (17.11)	125.72 (19.29)	<.001
Range	73–224	84–223	73–224	
**Diastolic blood pressure, nearest visit^c^, mm Hg**				
Mean (SD)	76.50 (10.57)	76.55 (10.42)	76.42 (10.77)	.64
Range	38–133	42–133	38–118	
**Ankle-brachial index**				
Mean (SD)	1.18 (0.14)	1.18 (0.13)	1.18 (0.15)	.04
Range	0.52–2.83	0.59–2.83	0.52–2.35	
**Ankle-brachial index category, n (%)**				
Low (≤0.9)	77 (1.4)	30 (0.9)	47 (2.0)	<.001
Normal (0.9–1.4)	5,269 (94.1)	3,074 (95.3)	2,195 (92.4)	
High (>1.4)	255 (4.6)	121 (3.8)	134 (5.6)	
**Body mass index, kg/m^2^**				
Mean (SD)	30.76 (6.77)	31.49 (6.73)	29.77 (6.68)	.004
Range	15.40–91.43	15.62–74.36	15.40–91.43	
**LDL, mg/dL**				
Mean (SD)	106.05 (31.73)	106.53 (31.21)	105.40 (32.41)	.18
Range	9.00–288.00	9.00–274.00	10.00–288.00	
**HDL, mg/dL**				
Mean +/− SD	48.38 (14.50)	48.19 (14.11)	48.64 (15.02)	.25
Range	12.00–146.00	16.00–138.00	12.00–146.00	
**Triglycerides, mg/dL^d^**				
Median	123.00	126.00	117.00	<.001
Range	2.00–5,323.00	2.00–5,323.00	7.00–1,757.00	
**Medical history, n (%)**				
History of myocardial infarction	103 (1.8)	49 (1.5)	54 (2.2)	.04
History of stroke	33 (0.6)	10 (0.3)	23 (0.9)	.002
Hypertension	1,916 (32.8)	1,082 (32.3)	834 (33.4)	.34
Diabetes	1,697 (29.0)	963 (28.7)	734 (29.4)	.49
Kidney disease	1,598 (27.3)	867 (25.9)	731 (29.3)	.004
**Smoking status, n (%)**				
Never smoked	1,941 (33.2)	1,099 (32.8)	842 (33.8)	.06
Previous smoker	1,668 (28.5)	997 (29.8)	671 (26.9)	
Current smoke	2,231 (38.2)	1,253 (37.4)	978 (39.3)	
**Alcohol consumption, n (%)**				
Never drank	764 (13.1)	429 (12.8)	335 (13.5)	.005
Previous drinker	2,111 (36.1)	1,271 (38.0)	840 (33.8)	
Current drinker	2,958 (50.6)	1,648 (49.2)	1,310 (52.7)	

Abbreviations: HDL, high-density lipoprotein cholesterol; LDL, low-density lipoprotein cholesterol.

a Strong Heart Study (26), Strong Heart Family Study (27).

b *t* tests used for continuous variables and χ^2^ used for categorical variables.

c The nearest visit is the study exam closest in time from the date of the 8th clinic blood pressure measurement.

d Wilcoxon test used.

**Figure 2:**
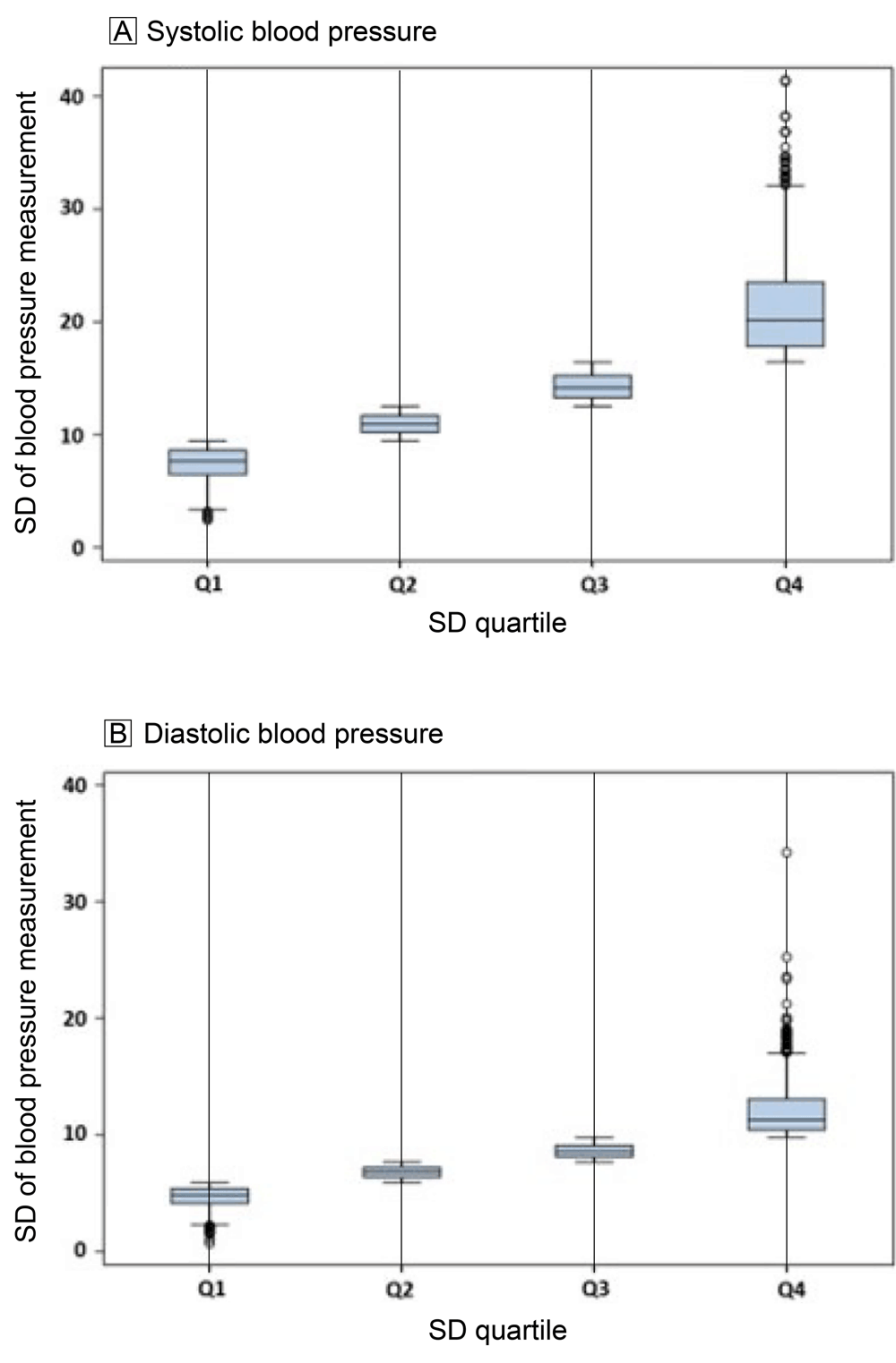
Box plots of the quartiles of the SD of systolic blood pressure and diastolic blood pressure for 8 nonurgent blood pressure measurements taken at clinic visits (urgent visits were defined as hospitalizations, emergency department visits, urgent care visits, ambulance trips, and pregnancy clinical visits and excluded) within a 5-year period closest to the dates of the Strong Heart Study (26) examination 3 (1997–1999) or the Strong Heart Family Study (27) examination 1 (2001–2003) of American Indians residing in in Arizona, Oklahoma, North Dakota, and South Dakota.

**Figure 3:**
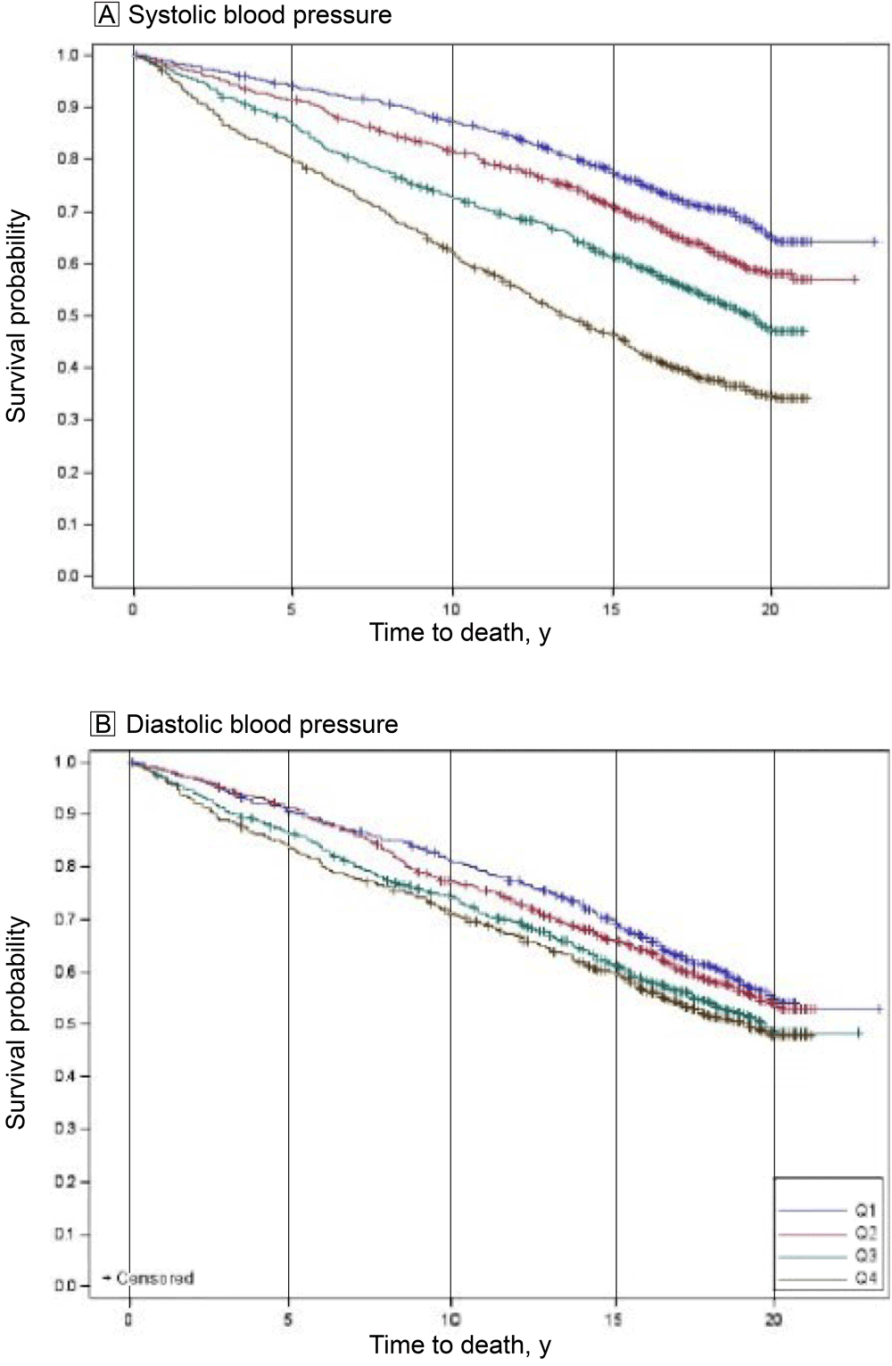
Survival curves for all-cause mortality, by quartiles, of the SD of systolic blood pressure (A) and diastolic blood pressure (B) for 8 blood pressure measurements taken at nonurgent clinic visits (urgent visits were defined as hospitalizations, emergency department visits, urgent care visits, ambulance trips, and pregnancy clinical visits and excluded) within 5 years, over 23 years of follow-up (1999–2022) for American Indians residing in Arizona, Oklahoma, North Dakota, and South Dakota.

We calculated the SBPSD and DBPSD quartiles and the mean, median, and ranges of the SBPSD and DBPSD for each quartile (Figure 2). For example, the medians of successive quartiles for SBPSD differ by 3.3, 3.2, and 6.0, and quartile 4 for SBPSD had a median value of 20.1 and a range of 16.4 to 41.4 mm Hg. We also conducted an initial analysis of the relationship of SBPSD and DBPSD as Kaplan–Meier Curves (Figure 3). The curves show a clear dose–response relationship between reduced survival from all-cause mortality with increasing quartiles of SBPSD. For DBPSD, quartiles 3 and 4 have significantly reduced survival versus quartile 1.

**Table:** 

Blood pressure, mmHg	Quartile 1	Quartile 2	Quartile 3	Quartile 4
Systolic blood pressure				
Minimum	2.4	9.4	12.5	16.4
25th Percentile	6.4	10.2	13.3	17.8
Median	7.6	10.9	14.1	20.1
75th Percentile	8.6	11.7	15.2	23.5
Maximum	9.4	12.5	16.4	41.4
Diastolic blood pressure				
Minimum	0.7	5.9	7.7	9.8
25th Percentile	4.1	6.4	8.1	10.4
Median	4.8	6.8	8.6	11.3
75th Percentile	5.4	7.2	9.1	13.1
Maximum	5.9	7.7	9.8	34.2

We summarized the effects of covariate adjustment on the hazard ratios (HRs) for SBPSD and DBPSD with additional covariates under Models 1 and 2 for all-cause mortality, CVD mortality, and MACE (Table 2). Details of the analyses for all covariates for both models are available (Appendix, Table 1A for SBPSD all-cause mortality and Appendix, Table 1B for DBPSD all-cause mortality; Appendix, tables 2A and 2B for CVD mortality; and Appendix, tables 3A and 3B for MACE).

**Table 2:** Blood Pressure Variation as a Prognostic Factor for All-Cause Mortality, Cardiovascular Disease Mortality, and Major Adverse Cardiovascular Events, With 8 Nonurgent^a^ Clinic Blood Pressure Measurements Within a 5-Year Period, by SD of Systolic Blood Pressure and Diastolic Blood Pressure Quartile, Combined Strong Heart Study Examination 3 (1997–1999) and Strong Heart Family Study Examination 1 (2001–2003)^b^

Event	Quartile	Unadjusted		Model 1^c^		Model 2^d^	
		HR (95% CI)	*P* value	HR (95% CI)	*P* value	HR (95% CI)	*P* value
**All-cause mortality**							
SBPSD	2	1.30 (1.07–1.58)	.009	1.09 (0.92–1.30)	.32	1.02 (0.85–1.23)	.81
	3	1.72 (1.42–2.09)	<.001	1.23 (1.04–1.51)	.02	1.17 (0.98–1.40)	.08
	4	2.51 (2.08–3.04)	<.001	1.51 (1.27–1.78)	<.001	1.35 (1.13 –1.61)	.001
DBPSD	2	1.07 (0.89–1.29)	.47	1.06 (0.91–1.24)	.46	1.01 (0.85–1.19)	.94
	3	1.32 (1.09–1.58)	.003	1.18 (1.01–1.38)	.04	1.17 (0.99–1.38)	.06
	4	1.40 (1.16–1.68)	<.001	1.26 (1.08–1.47)	.004	1.15 (0.97–1.36)	.10
**Cardiovascular disease mortality**							
SBPSD	2	1.68 (1.20–2.37)	.003	1.39 (1.01–1.91)	.05	1.24 (0.87–1.76)	.24
	3	1.82 (1.30–2.57)	<.001	1.28 (0.93–1.76)	.13	1.17 (0.82–1.66)	.38
	4	3.73 (2.70–5.13)	<.001	2.09 (1.55–2.83)	<.001	1.81 (1.29–2.53)	<.001
DBPSD	2	0.93 (0.69–1.26)	.65	0.96 (0.73–1.26)	.76	0.92 (0.68–1.26)	.62
	3	1.27 (0.95–1.71)	.11	1.19 (0.91–1.56)	.20	1.13 (0.84–1.52)	.43
	4	1.39 (1.04–1.86)	.03	1.30 (1.00–1.69)	.05	1.22 (0.91 –1.65)	.18
**Major adverse cardiovascular events**							
SBPSD	2	1.59 (1.22–2.07)	<.001	1.41 (1.11–1.78)	.004	1.36 (1.05–1.77)	.02
	3	1.49 (1.13–1.95)	.004	1.09 (0.86–1.39)	.47	1.06 (0.81–1.39)	.65
	4	2.53 (1.95–3.28)	<.001	1.49 (1.18–1.88)	<.001	1.39 (1.07–1.80)	.01
DBPSD	2	0.99 (0.77–1.28)	.97	1.02 (0.82–1.27)	.84	0.98 (0.78–1.25)	.90
	3	1.22 (0.96–1.57)	.11	1.19 (0.96–1.47)	.11	1.10 (0.87–1.39)	.45
	4	1.24 (0.97–1.60)	.08	1.18 (0.95–1.46)	.13	1.11 (0.87–1.40)	.41

Abbreviations: DBPSD, SD of diastolic blood pressure; HDL, high-density lipoprotein cholesterol; HR, hazard ratio; LDL, low-density lipoprotein cholesterol; SBPSD, SD of systolic blood pressure.

a Excluded hospitalizations, emergency room visits, urgent care visits, ambulance trips, and pregnancy clinical visits.

b Strong Heart Study (26), Strong Heart Family Study (27).

c Shared frailty Cox proportional hazards model adjusted for cohort, center, age, sex, and systolic blood pressure/diastolic blood pressure, as appropriate.

d Shared frailty Cox proportional hazards model adjusted for Model 1 and hypertension treatment, diabetes, body mass index, current smoking, current drinking, LDL, HDL, triglycerides kidney disease, ankle/brachial index, prevalent myocardial infarction and stroke (not for all-cause mortality), and interaction of prevalent systolic blood pressure or diastolic blood pressure and hypertension treatment.

Unadjusted analyses for all-cause mortality confirm the results of the Kaplan–Meier analysis of stronger relationships with each quartile of SBPSD (Table 2). Adjustment in Model 1 reduced the HRs but maintained significant effects for quartiles 3 and 4. Adjustment under Model 2 further reduced the HRs so that only quartile 4 remained significant (HR = 1.35; 95% CI, 1.13–1.61).

For DBPSD, unadjusted data show significant relationships for quartiles 3 and 4 with all-cause mortality. Model 1 results provided HRs that remained significant for quartile 3 (HR = 1.18; 95% CI, 1.01–1.38) and quartile 4 (HR = 1.26; 95% CI, 1.08–1.47). Adjustment under Model 2 showed attenuation of the HR point estimates, and they were no longer significant.

Similar analyses for CVD mortality showed results that were significant for quartile 2 and quartile 4 for SBPSD under Model 1. Results remained significant and substantial for quartile 4 under Model 2 (HR = 1.81; 95% CI, 1.29–2.53). For DBPSD, quartile 4 was significant under Model 1 (HR = 1.30; 95% CI, 1.00–1.69) but was no longer significant under Model 2.

Finally, the results for SBPSD for MACE show significant HRs for quartile 2, quartile 3, and quartile 4 in unadjusted analyses, remain significant for quartile 2 and quartile 4 under Model 1, and are significant for quartile 2 (HR=1.36; 95% CI, 1.05–1.77) and quartile 4 (HR = 1.39; 95% CI, 1.07–1.80) under Model 2. Results for DBPSD show no significant relationship with MACE before or after adjustment.

It is worth noting that all the *P *values for diabetes, renal disease, and prevalent MI and stroke are *P* <.05 and for ABI are *P* <.07 for all 3 endpoints for both SBPSD and DBPSD (Appendix, Tables 1A–3B) suggesting these morbidity measures play a significant role in reducing the HRs and their significance for BPV for these endpoints (Table 2, Appendix, Tables 1A–3B).

We performed a sensitivity analysis to investigate potential differences in results for individuals who have nonurgent clinic visits clustered over a shorter interval. The time interval for collection of 8 blood pressures was dichotomized at less than or equal to 1 year (n = 1,232 [36.8%]) versus 1-to-5 years (n = 2,120 [63.2%]). We found similar trends in both groups but stronger results for those in the 1-to-5-year group, particularly for Model 2 results for SBPSD. Findings were also significant for quartiles 3 and 4 for all-cause mortality, for quartiles 2, 3, and 4 for cardiovascular mortality, and for MACE. Additional stratified analyses related to the covariates in the model were assessed by testing interactions in the model, and none was found to be significant.

## Discussion

Ours is the first study to assess the relationship of BPV to all-cause mortality, CVD mortality, and MACE in American Indians. Our study confirmed significant prognostic value for SBPSD with all-cause mortality, CVD mortality, and MACE, primarily for the highest quartile of SBPSD. Like many previous analyses of non-American Indian samples, results for DBPSD were less compelling, with significant results for quartile 4 under Model 1 for all-cause mortality and CVD mortality that did not survive the adjustment for CVD risk factors under Model 2. Findings were strongest for all-cause mortality and CVD mortality, then MACE, and were probably affected by the number of events available for analysis and the exclusions of those with prevalent disease in the case of the MACE analyses. The differences in the medians of SBPSD between quartile 1 and quartile 2, quartile 2 and quartile 3, and quartile 3 and quartile 4 were 3.3, 3.2, and 6.0 mm Hg, respectively. Thus, it is not surprising that significant findings were primarily in quartiles 3 and 4, given that the pooled results for 1 standardized log hazard ratio of SBPSD would represent 5.7 mm Hg for this study as described in the methods of Stevens and colleagues (31). These findings support the conclusion that there is little evidence of racial- and ethnicity-specific differences in the effects of BPV on the outcomes addressed.

The mechanisms linking BPV to all-cause mortality, CVD mortality, and MACE are autonomic dysfunction, endothelial dysfunction, atherosclerosis, vascular stiffness, aortic distensibility, diastolic dysfunction, subclinical inflammation, and cognitive decline. BPV may also reflect seasonal effects, measurement errors, antihypertensive treatment effects, and medication adherence (21,24,32).

It might be argued that much of the effect of BPV on all-cause and CVD morbidity and mortality may be via a wide array of end organ damage (33). In this analysis, end organ damage of BPV may be captured more proximally by the covariates used for adjustment in Model 2: history of CVD, peripheral vascular disease, diabetes, or kidney disease. As noted in our introduction, the literature is extensive linking SD and other measures of BPV to clinical cardiovascular disease (2,4–17) and subclinical CVD (19,21,22). In relation to diabetes, visit-to-visit BPV measured by average real variability for both SBP and DBP was a significant prognostic indicator for the development of type 2 diabetes in a Chinese cohort over a 16-year follow-up (20). In regard to kidney disease, in a large sample of US veterans, SBPSD based on 8 or more outpatient blood pressure measurements was significantly associated with end stage renal disease in a dose responsive way for quartiles 2, 3, and 4 compared with quartile 1 of SBPSD (5). Multiple studies have shown BPV is a prognostic indicator for albuminuria (34,35). Thus, our Model 2 adjustments for prevalent CVD, ABI, diabetes, and kidney disease may represent over-adjustment, and the results for BPV after adjustment in Model 1 may be more representative of the underlying prognostic effects of BPV on the endpoints. In this interpretation, DBPSD would be significantly related to both all-cause mortality (quartiles 3 and 4) and CVD mortality (quartile 4). The HRs for the above covariates were highly significant in Model 2 adjustments for SBPSD and DBPSD for all-cause mortality, cardiovascular mortality, and MACE. The decline and reduced significance in HRs, particularly for SBPSD, from unadjusted to Model 1 to Model 2 are certainly compatible with the foregoing interpretation. However, the fact that SBPSD endures these adjustments, particularly for quartile 4 for all-cause mortality and cardiovascular mortality, suggests the potential strength of this measure as a prognostic indicator. The use of causal mediation analysis to investigate the potential for indirect effects of BPV on outcomes may make more targeted mechanistic investigations possible.

Results of the sensitivity analysis dichotomizing the sample at 1 year strengthened the results for the two-thirds of the sample in the 1-to 5-year group, suggesting that those with 8 blood pressures taken in nonurgent clinic visits in less than 1 year may be different from those requiring a longer time to reach this frequency. Further research is needed to understand the nuances of the group differences.

We have attempted to address many of the statistical and methodological challenges described in the literature. First, raw blood pressure data used for these analyses were limited to nonurgent visits to a wide array of medical care providers without the benefit of standardized blood pressure protocols normally found in epidemiological studies. As such, they represent real-world blood pressure levels and variability as might be found in most large medical care systems. Second, the number of blood pressures required for eligibility was set at 8 based on a tradeoff between a larger sample size, longer follow-up for events, and reduced secular blood pressure effects associated with fewer, rather than more visits. The literature suggests stability occurs with a minimum of 6 measurements (7). Third, the calculation of BPV focused on the SD as the measure of variability of visit-to-visit blood pressures because it is the most frequently cited measure of blood pressure variability (31). Fourth, the time interval over which the blood pressure measures were taken was limited to 5 years as in other studies (8,10,13), and most measures for this analysis were taken within a 2-year interval to minimize secular changes in blood pressure and consequently blood pressure variability. Fifth, analyses for each endpoint included the level of blood pressure in both adjusted models to account for its effect on blood pressure variability. Sixth, the follow-up for events extended to 2 decades to minimize the influence of short-term effects. Finally, only the first blood pressure taken on each visit was used to maintain comparability of the measures across participants and visits. 

This study is not without limitations. First, we used blood pressure recorded in a variety of clinic visits, which may therefore reflect greater variability than those collected in a single setting, or following standardized measurement protocols. However, these measurements are likely to be representative of the range of blood pressures that would be found in real-world clinical settings, including electronic health records data from large health care facilities. Also, this type of variability would reduce the observability of associations (Type II error), which would not affect any detected or reported associations. Second, this study was conducted in a ell-characterized cohort of participants, many of whom reside in a rural setting and with a unique risk factor profile; results should be interpreted with caution for generalizability to other populations. As evidence of BPV as a potential risk factor continues to mount, we need to determine which measures of BPV are associated with adverse events and settle on their definitions. In a world of significant progress in data mining, the incorporation of these measures, once defined, into large electronic health record systems appears relatively straightforward. The next challenge will be to determine whether it is possible to modify BPV with existing or new treatments, and finally, whether reduction of BPV will affect subsequent morbidity and/or mortality.

Our analysis offers the first look at the prognostic value of blood pressure variability for all-cause mortality, cardiovascular mortality, and MACE in American Indians. It demonstrates clear and significant differences in survival in a dose responsive way for quartiles of SBPSD, and for quartiles of DBPSD (beginning in quartile 3). After adjustment for cardiovascular risk factors, significant differences for BPV with all-cause mortality were persistent for quartile 4 for SBPSD. Finally, the results for CVD mortality and MACE are not as consistent in dose response or in final significance in adjusted models, particularly for DBPSD, and may reflect lack of effect, smaller numbers of events, the vagaries of sample variability, or missing covariates.

## Appendix

**Appendix Table 1A:** Association Between All-Cause Mortality and Systolic Blood Pressure SD Quartiles of 8 Nonurgent^a^ Clinic Visits Within a 5-Year Period

Variable	Unadjusted			Model 1 adjusted^b^			Model 2 adjusted^c^		
	Hazard ratio	95% CI	*P* value	Hazard ratio	95% CI	*P* value	Hazard ratio	95% CI	*P* value
**Systolic blood pressure SD quartiles (reference, quartile 1)**									
2	1.30	1.07–1.58	<.001	1.09	0.92–1.30	.32	1.02	0.85–1.23	.81
3	1.72	1.42–2.09	<.001	1.23	1.04–1.46	.02	1.17	0.98–1.40	.08
4	2.51	2.08–3.04	<.001	1.51	1.27–1.78	<.001	1.35	1.13–1.61	.001
**Strong Heart Study (** 26 **) cohort (reference, Strong Heart Family Study [** 27 **])**									
Strong Heart Study	4.72	4.14–5.39	<.001	1.29	1.04–1.60	.02	1.27	1.00–1.63	.05
**Sex (reference, female)**									
Male	1.45	1.26–1.65	<.001	0.72	0.64–0.81	<.001	0.74	0.65–0.84	<.001
**Strong Heart Study site (reference, Oklahoma)**									
Arizona	0.87	0.69–1.10	0.25	1.15	0.96–1.37	0.13	1.10	0.89–1.35	.38
South Dakota	1.33	1.12–1.57	<.001	1.38	1.22–1.57	<.001	1.34	1.17–1.54	<.001
**Age at 8th blood pressure measurement, y**	1.06	1.06–1.07	<.001	1.06	1.05–1.06	<.001	1.06	1.05–1.07	<.001
**Baseline systolic blood pressure, mm Hg (continuous)**	1.02	1.01–1.02	<.001	1.01	1.00–1.01	.004	1.00	0.99–1.01	.94
**Hypertension treatment (reference, no)**									
Yes	1.86	1.62–2.13	<.001	NA	NA	NA	0.51	0.21–1.22	.13
**Diabetes (reference, no)**									
Yes	2.15	1.89–2.45	<.001	NA	NA	NA	1.66	1.46–1.88	<.001
**Body mass index (kg/m^2^) baseline (continuous)**	1.80	1.50–2.61	<.001	NA	NA	NA	1.00	0.99–1.01	.98
**Current smoker (reference, no)**									
Yes	1.15	1.01–1.31	.03	NA	NA	NA	1.28	1.12–1.45	<.001
**Current drinker (reference, no)**									
Yes	0.82	0.72–0.93	.003	NA	NA	NA	1.12	0.98–1.27	.10
**LDL, mg/dL (reference, <100 mg/dL)**									
100-129	0.91	0.78–1.06	.24	NA	NA	NA	0.84	0.73–0.97	.02
130-159	0.95	0.78–1.14	.57	NA	NA	NA	0.83	0.70–0.98	.03
160-189	0.96	0.71–1.30	.82	NA	NA	NA	0.74	0.57–0.95	.02
≥190	1.04	0.63–1.73	.87	NA	NA	NA	1.01	0.66–1.54	.97
**HDL, mg/dL (reference: men, <40 mg/dL; women, <50 mg/dL)**									
Men, 40-59, women 51-59	0.73	0.64–0.83	<.001	NA	NA	NA	1.01	0.87–1.18	.88
Men ≥60, women ≥60	0.60	0.52–0.71	<.001	NA	NA	NA	1.08	0.89–1.31	.44
**Triglycerides, mg/dL (reference, <150 mg/dL)**									
150–199	1.03	0.87–1.23	.71	NA	NA	NA	0.96	0.82–1.12	.61
200–499	1.06	0.90–1.25	.51	NA	NA	NA	0.83	0.71–0.97	.02
≥500	1.10	0.70–1.71	.60	NA	NA	NA	0.85	0.57–1.27	.42
**Kidney disease (reference, no)**									
Yes	2.37	1.99–2.82	<.001	NA	NA	NA	1.27	1.08–1.50	.005
**Ankle/brachial index (reference, >0.9 to**≤1**.4)**									
≤0.9 or >1.4	2.01	1.57–2.56	<.001	NA	NA	NA	1.22	0.99–1.51	.06
**Prevalent myocardial infarction or stroke (reference, no)**									
Yes	3.06	2.32–4.04	<.001	NA	NA	NA	1.55	1.22–1.99	<.001

Abbreviations: NA, not applicable; HDL, high-density lipoprotein cholesterol; LDL, low-density lipoprotein cholesterol.

^a^ Excluded hospitalizations, emergency room visits, urgent care visits, ambulance trips, and pregnancy clinical visits.

^b^ Shared frailty Cox proportional hazards model adjusted for cohort, center, age, sex, and systolic blood pressure/diastolic blood pressure, as appropriate.

^c^ Shared frailty Cox proportional hazards model adjusted for cohort, sex, center, age, baseline systolic blood pressure, hypertension treatment, diabetes mellitus, body mass index, current smoking, current drinking, LDL, HDL, triglycerides, kidney disease, ankle/brachial index, prevalent myocardial infarction and stroke interaction of systolic blood pressure, and hypertension treatment.

**Appendix Table 1B:** Association Between Diastolic Blood Pressure SD Quartiles of 8 Nonurgent^a^ Clinic Visits Within a 5-Year Period and All-Cause Mortality

Variable	Unadjusted			Model 1 Adjusted^b^			Model 2 Adjusted^c^		
	Hazard ratio	95% CI	*P* value	Hazard ratio	95% CI	*P* value	Hazard ratio	95% CI	*P* value
**Diastolic blood pressure SD quartiles (reference, quartile 1)**									
2	1.07	0.89–1.29	.72	1.06	0.91–1.24	.46	1.01	0.85–1.19	.94
3	1.32	1.09–1.58	.003	1.18	1.01–1.38	.04	1.17	0.99–1.38	.06
4	1.40	1.16–1.68	<.001	1.26	1.08–1.47	.004	1.15	0.97–1.36	.10
**Strong Heart Study (** 26 **) cohort (reference, Strong Heart Family Study [** 27 **])**	4.72	4.14–5.39	<.001	1.29	1.05–1.60	.02	1.27	1.00–1.62	.05
**Sex (reference, female)**									
Male	1.45	1.26–1.65	<.001	0.71	0.63–0.80	<.001	0.74	0.65–0.84	<.001
**Strong Heart Study site (reference, Oklahoma)**									
Arizona	0.87	0.69–1.10	.25	1.13	0.94–1.35	.19	1.06	0.86–1.30	.58
South Dakota	1.33	1.12–1.57	<.001	1.36	1.20–1.55	<.001	1.33	1.16–1.52	<.001
**Age at eighth blood pressure measurement, y (continuous)**	1.06	1.06–1.07	<.001	1.06	1.05–1.07	<.001	1.06	1.05–1.07	<.001
**Baseline diastolic blood pressure, mm Hg (continuous)**	1.02	1.01–1.02	<.001	1.00	0.99 – 1.00	.13	1.00	0.99–1.01	.23
**Hypertension treatment (reference, no)**									
Yes	1.86	1.62–2.13	<.001	NA	NA	NA	0.77	0.32–1.88	.57
**Diabetes (reference, no)**									
Yes	2.15	1.89–2.45	<.001	NA	NA	NA	1.68	1.48–1.91	<.001
**Body mass index (kg/m^2^) baseline (continuous)**	1.80	1.50–2.61	<.001	NA	NA	NA	1.00	0.99–1.01	.90
**Current smoker (reference, no)**									
Yes	1.15	1.01–1.31	.03	NA	NA	NA	1.28	1.12–1.45	<.001
**Current drinker (reference, no)**									
Yes	0.82	0.72–0.93	.003	NA	NA	NA	1.12	0.99–1.28	.07
**LDL, mg/dL (reference, ≤100 mg/dL)**									
100–129	0.91	0.78–1.06	.24	NA	NA	NA	0.84	0.73–0.97	.02
130–159	0.95	0.78–1.14	.57	NA	NA	NA	0.83	0.70–0.99	.04
160–189	0.96	0.71–1.30	.82	NA	NA	NA	0.77	0.60–0.98	.04
≥190 mg/dL	1.04	0.63–1.73	.87	NA	NA	NA	1.04	0.68–1.58	.87
**HDL (reference, men, <40; women, <50)**									
Men 40–59, omen 51–59	0.73	0.64–0.83	<.001	NA	NA	NA	1.01	0.87–1.18	.89
Men, ≥60; women, ≥60	0.60	0.52–0.71	<.001	NA	NA	NA	1.09	0.90–1.32	.40
**Triglycerides, mg/dL (reference, ≥150 mg/dL)**									
150–199 mg/dL	1.03	0.87–1.23	.71	NA	NA	NA	0.96	0.82–1.13	.64
200–499 mg/dL	1.06	0.90–1.25	.51	NA	NA	NA	0.84	0.72–0.98	.03
≥500 mg/dL	1.10	0.70–1.71	.69	NA	NA	NA	0.88	0.59–1.31	.53
**Kidney disease (reference, no)**									
Yes	2.37	1.99–2.82	<.001	NA	NA	NA	1.30	1.10–1.53	.002
**Ankle/brachial index (reference**≥**0.9 to ≤1.4)**									
≤0.9 or >1.4	2.01	1.57–2.56	<.001	NA	NA	NA	1.23	0.99–1.51	.06
**Prevalent myocardial infarction or stroke (reference, no)**									
Yes	3.06	2.32–4.04	<.001	NA	NA	NA	1.52	1.19–1.94	<.001

Abbreviations: NA, not applicable; HDL, high-density lipoprotein cholesterol; LDL, low-density lipoprotein cholesterol.

^a^ Excluded hospitalizations, emergency room visits, urgent care visits, ambulance trips, and pregnancy clinical visits.

^b^ Shared frailty Cox proportional hazards model adjusted for cohort, center, age, sex, and systolic blood pressure/diastolic blood pressure, as appropriate.

^c^ Shared frailty Cox proportional hazards model adjusted for cohort, sex, center, age, baseline systolic blood pressure, hypertension treatment, diabetes, body mass index, current smoking, current drinking, LDL, HDL, triglycerides, kidney disease, ankle/brachial index, prevalent myocardial infarction and stroke, interaction of systolic blood pressure, and hypertension treatment.

**Appendix Table 2A:** Association Between Systolic Blood Pressure SD Quartiles of 8 Nonurgent^a^ Clinic Visits Within a 5-Year Period and Cardiovascular Mortality

Variable	Unadjusted			Model 1 adjusted^b^			Model 2 adjusted^c^		
	Hazard ratio	95% CI	*P* value	Hazard ratio	95% CI	*P* value	Hazard ratio	95% CI	*P* value
**Systolic blood pressure SD quartiles (reference, quarter 1)**									
2	1.68	1.20–2.37	.003	1.39	1.01–1.91	.05	1.24	0.87–1.76	.24
3	1.82	1.30–2.57	<.001	1.28	0.93–1.76	.13	1.17	0.82–1.66	.38
4	3.73	2.70–5.13	<.001	2.09	1.55–2.83	<.001	1.81	1.29–2.53	<.001
**Strong Heart Study (** 26 **) cohort (reference, Strong Heart Family Study (** 27 **)**									
Strong Heart Study	7.24	5.52–9.49	<.001	1.60	1.12–2.30	.01	1.81	1.15–2.85	.01
Sex (reference, female)									
Male	1.57	1.27–1.94	<.001	0.66	0.54–0.80	<.001	0.6	0.51–0.81	<.001
**Strong Heart Study site (reference, Oklahoma)**									
Arizona	0.61	0.41–0.90	0.01	0.82	0.58–1.13	.23	0.79	0.52–1.19	.25
South Dakota	1.35	1.05–1.73	0.02	1.41	1.15–1.73	.001	1.44	1.14–1.83	.002
**Age at eighth blood pressure measurement, y (continuous)**	1.08	1.07–1.09	<.001	1.07	1.05–1.08	<.001	1.06	1.05–1.07	<.001
**Baseline systolic blood pressure, mm Hg (continuous)**	1.02	1.02–1.03	<.001	1.01	1.00–1.01	.01	1.00	0.99–1.01	.63
**Hypertension treatment (reference, no)**									
Yes	2.67	2.16–3.30	<.001	NA	NA	NA	0.94	0.12–2.68	.48
**Diabetes (reference, no)**									
Yes	3.24	2.61–4.02	<.001	NA	NA	NA	2.49	1.97–3.13	<.001
Body mass index (kg/m^2^) baseline (continuous)	0.99	0.97–1.00	.03	NA	NA	NA	0.99	0.97–1.01	.46
**Current smoker (reference, no)**									
Yes	1.08	0.88–1.34	.46	NA	NA	NA	1.30	1.03–1.63	.03
**Current drinker (reference, no)**									
Yes	0.62	0.50–0.76	<.001	NA	NA	NA	0.89	0.70–1.12	.30
**LDL l, mg/dL (reference, ≥100 mg/dL)**									
100–129	1.10	0.85–1.42	.48	NA	NA	NA	0.95	0.73–1.24	.72
130–159	1.26	0.94–1.70	.12	NA	NA	NA	1.02	0.75–1.38	.90
160–189	1.35	0.86–2.11	.19	NA	NA	NA	0.82	0.53–1.27	.38
≥190	1.56	0.75–3.23	.23	NA	NA	NA	1.33	0.65–2.76	.44
**HDL mg/dL(reference, men <40; women <50 mg/dL)**									
Men 40–59, women 51–59	0.72	0.58–0.90	.003	NA	NA	NA	1.16	0.88–1.52	.30
Men ≥60, women ≥60	0.51	0.38–0.68	<.001	NA	NA	NA	1.26	0.87–1.83	.21
**Triglycerides (reference, <150 mg/dL)**									
150–199 mg/dL	1.33	1.01–1.75	.04	NA	NA	NA	1.17	0.88–1.55	.28
200–499 mg/dL	1.51	1.17–1.95	.002	NA	NA	NA	1.07	0.82–1.41	.61
≥500 mg/dL	1.09	0.51–2.30	.83	NA	NA	NA	0.93	0.45–1.90	.84
**Kidney disease (reference, no)**									
Yes	3.40	2.62–4.40	<.001	NA	NA	NA	1.51	1.15–1.99	.003
**Ankle/brachial index (reference, >0.9 to **≤**1.4)**									
**≤**0.9 or >1.4	2.68	1.87–3.85	<.001	NA	NA	NA	1.49	1.05–2.10	.03
**Prevalent myocardial infarction or stroke (reference, no)**									
Yes	5.73	3.99–8.22	<.001	NA	NA	NA	2.47	1.73–3.52	<.001

Abbreviations: NA, not applicable; LDL, low-density lipoprotein cholesterol; HDL, high-density lipoprotein cholesterol.

^a^ Excluded hospitalizations, emergency room visits, urgent care visits, ambulance trips, and pregnancy clinical visits.

^b^ Shared frailty Cox proportional hazards model adjusted for cohort, center, age, sex, and systolic blood pressure/diastolic blood pressure, as appropriate.

^c^ Shared frailty Cox proportional hazards model adjusted for cohort, sex, center, age, baseline systolic blood pressure, hypertension treatment, diabetes, body mass index, current smoking, current drinking, LDL, HDL, triglycerides, kidney disease, ankle/brachial index, prevalent myocardial infarction and stroke, interaction of systolic blood pressure, and hypertension treatment.

**Appendix Table 2B:** Association Between Diastolic Blood Pressure SD Quartiles of 8 Nonurgent^a^ Clinic Visits Within a 5-Year Period and Cardiovascular Mortality

Variable	Unadjusted			Model 1, adjusted^b^			Model 2, adjusted^c^		
	Hazard ratio	95% CI	*P* value	Hazard ratio	95% CI	*P* value	Hazard ratio	95% CI	*P* value
**Diastolic blood pressure SD quartiles (reference, quartile 1)**									
2	0.93	0.69–1.26	.65	0.96	0.73–1.26	.76	0.92	0.68–1.26	.62
3	1.27	0.95–1.71	.11	1.19	0.91–1.56	.20	1.13	0.84–1.52	.43
4	1.39	1.04–1.86	.03	1.30	1.00–1.69	.05	1.22	0.91–1.65	.18
**Strong Heart Study (** 26 **) cohort (reference, Strong Heart Family Study (** 27 **)**									
Strong Heart Study	7.24	5.52–9.49	<.001	1.63	1.14–2.34	.007	1.85	1.18–2.90	.007
**Sex (reference, female)**									
Male	1.57	1.27–1.94	<.001	0.66	0.55–0.81	<.001	0.64	0.51–0.81	<.001
**Strong Heart Study site (reference, Oklahoma)**									
Arizona	0.61	0.41–0.90	.01	0.79	0.56–1.09	.15	0.74	0.49–1.11	.15
South Dakota	1.35	1.05–1.73	.02	1.37	1.12–1.69	.002	1.44	1.14–1.82	.002
**Age at eighth blood pressure measurement, y**	1.08	1.07–1.09	<.001	1.07	1.06–1.08	<.001	1.06	1.05–1.08	<.001
**Baseline diastolic blood pressure, mm Hg (continuous)**	1.02	1.02–1.03	<.001	0.99	0.98 – 1.00	0.15	0.99	0.97–1.00	.12
**Hypertension treatment (reference, no)**									
Yes	2.67	2.16–3.30	<.001	NA	NA	NA	0.98	0.16–3.98	.79
**Diabetes (reference, no)**									
Yes	3.24	2.61–4.02	<.001	NA	NA	NA	2.50	1.98–3.14	<.001
**Body mass index (kg/m^2^**) **baseline (continuous)**	0.99	0.97–1.00	.03	NA	NA	NA	0.99	0.98–1.01	.58
**Current smoker (reference, no)**									
Yes	1.08	0.88–1.34	.46	NA	NA	NA	1.31	1.04–1.64	.02
**Current drinker (reference, no)**									
Yes	0.62	0.50–0.76	<.001	NA	NA	NA	0.91	0.73–1.14	.41
**LDL (reference, 100 mg/dL)**									
100–129	1.10	0.85–1.42	.48	NA	NA	NA	0.96	0.74–1.25	.76
130–159	1.26	0.94–1.70	.12	NA	NA	NA	1.05	0.77–1.42	.76
160–189	1.35	0.86–2.11	.19	NA	NA	NA	0.88	0.57–1.36	.57
≥190	1.56	0.75–3.23	.23	NA	NA	NA	1.39	0.67–2.87	.37
**HDL (reference, men, <40; women, <50)**									
Men 40–59, women 51–59	0.72	0.58–0.90	.003	NA	NA	NA	1.15	0.87–1.52	.31
Men ≥60, Women ≥60	0.51	0.38–0.68	<.001	NA	NA	NA	1.28	0.88–1.84	.19
**Triglycerides (reference, ≥150 mg/dL)**									
150–199 mg/dL	1.33	1.01–1.75	.04	NA	NA	NA	1.17	0.88–1.54	.28
200–499 mg/dL	1.51	1.17–1.95	.002	NA	NA	NA	1.10	0.84–1.45	.48
≥500 mg/dL	1.09	0.51–2.30	.83	NA	NA	NA	1.00	0.49–2.04	.99
**Kidney disease (reference, no)**									
Yes	3.40	2.62–4.40	<.001	NA	NA	NA	1.58	1.21–2.08	.001
**Ankle/brachial index (reference, >0.9 to ≤1.4)**									
**≤**0.9 or >1.4	2.68	1.87–3.85	<.001	NA	NA	NA	1.51	1.07–2.13	.02
**Prevalent myocardial infarction or stroke (reference, no)**									
Yes	5.73	3.99–8.22	<.001	NA	NA	NA	2.39	1.68–3.39	<.001

Abbreviations: NA, not applicable; LDL, low-density lipoprotein cholesterol; HDL, high-density lipoprotein cholesterol.

^a^ Excluded hospitalizations, emergency room visits, urgent care visits, ambulance trips, and pregnancy clinical visits.

^b^ Shared frailty Cox proportional hazards model adjusted for cohort, center, age, sex, and systolic blood pressure/diastolic blood pressure, as appropriate.

^c^ Shared frailty Cox proportional hazards model adjusted for cohort, sex, center, age, baseline diastolic blood pressure, hypertension treatment, diabetes, body mass index, current smoking, current drinking, LDL, HDL, triglycerides, kidney disease, ankle/brachial index, prevalent myocardial infarction and stroke, interaction of systolic blood pressure, and hypertension treatment.

**Appendix Table 3A:** Association Between Systolic Blood Pressure SD Quartiles of 8 Nonurgent^a^ Clinic Visits Within a 5-Year Period and Major Adverse Cardiovascular Events

Variable	Unadjusted			Model 1, adjusted^b^			Model 2, adjusted^c^		
	Hazard ratio	95% CI	*P* value	Hazard ratio	95% CI	*P* value	Hazard ratio	95% CI	*P* value
**Diastolic blood pressure SD quartiles (reference, quartile 1)**									
2	1.59	1.22–2.07	<.001	1.41	1.11–1.78	.004	1.36	1.05–1.77	.02
3	1.49	1.13–1.95	.004	1.09	0.86–1.39	.47	1.06	0.81–1.39	.65
4	2.53	1.95–3.28	<.001	1.49	1.18–1.88	<.001	1.39	1.07–1.80	.01
**Strong Heart Study (** 26 **) cohort (reference, Strong Heart Family Study (** 27 **)**									
Strong Heart Study	4.84	3.99–5.86	<.001	1.35	1.05–1.74	.02	1.38	0.98–1.95	.06
**Sex (reference, female)**									
Male	1.69	1.41–2.03	<.001	0.64	0.55–0.75	<.001	0.62	0.52–0.75	<.001
**Strong Heart Study site (reference, Oklahoma)**									
Arizona	0.75	0.53–1.07	.11	0.92	0.69–1.22	.55	0.91	0.65–1.28	.60
South Dakota	2.34	1.87–2.94	<.001	2.25	1.90–2.67	<.001	2.41	1.98–2.94	<.001
**Age at eighth blood pressure, y (continuous)**	1.06	1.05–1.07	<.001	1.05	1.05–1.06	<.001	1.05	1.04–1.06	<.001
**Baseline systolic blood pressure, mm Hg (continuous)**	1.02	1.01–1.02	<.001	1.01	1.00–1.01	<.001	1.00	1.00–1.01	.20
**Hypertension treatment (reference, no)**									
Yes	2.22	1.84–2.67	<.001	NA	NA	NA	0.95	0.27–3.28	.93
**Diabetes (reference, no)**									
Yes	2.64	2.20–3.16	<.001	NA	NA	NA	1.97	1.64–2.36	<.001
**Body mass index (kg/m^2^**) **baseline (continuous)**	0.99	0.99–1.00	.02	NA	NA	NA	0.99	0.98–1.01	.48
**Current smoker (reference, no)**									
Yes	1.30	1.09–1.56	.003	NA	NA	NA	1.41	1.18–1.69	.001
**Current drinker (reference, no)**									
Yes	0.69	0.58–0.83	<.001	NA	NA	NA	0.84	0.70–1.02	.07
**LDL (reference,<100 mg/dL)**									
100–129	1.18	0.94–1.46	.15	NA	NA	NA	1.08	0.87–1.33	.51
130–159	1.73	1.35–2.22	<.001	NA	NA	NA	1.32	1.04–1.68	.02
160–189	1.60	1.08–2.36	.02	NA	NA	NA	0.95	0.67–1.36	.79
≥190	1.18	0.59–2.36	.63	NA	NA	NA	0.90	0.47–1.74	.75
**HDL (reference, men <40; women <50)**									
Men 40–59, women 51–59	0.72	0.58–0.90	.003	NA	NA	NA	1.05	0.84–1.30	.68
Men ≥60, women ≥60	0.51	0.38–0.68	<.001	NA	NA	NA	0.99	0.74–1.32	.95
**Triglycerides (reference, <150 mg/dL)**									
150–199	1.31	1.04–1.65	.02	NA	NA	NA	1.10	0.88–1.38	.40
200–499	1.37	1.10–1.72	.005	NA	NA	NA	0.98	0.78–1.22	.85
≥500	1.63	0.91–2.91	.10	NA	NA	NA	1.24	0.71–2.16	.46
**Kidney disease (reference, no)**									
Yes	2.68	2.12–3.39	<.001	NA	NA	NA	1.42	1.12–1.79	.003
**Ankle/brachia index (reference, >0.9 to ≤1.4)**									
≤0.9 or >1.4	2.11	1.51–2.95	<.001	NA	NA	NA	1.33	0.98–1.82	.07

Abbreviations: NA, not applicable; LDL, low-density lipoprotein cholesterol; HDL, high-density lipoprotein cholesterol.

^a^ Excluded hospitalizations, emergency room visits, urgent care visits, ambulance trips, and pregnancy clinical visits.

^b^ Shared frailty Cox proportional hazards model adjusted for cohort, center, age, sex, and systolic blood pressure/diastolic blood pressure, as appropriate.

^c^ Shared frailty Cox proportional hazards model adjusted for cohort, sex, center, age, baseline systolic blood pressure, hypertension treatment, diabetes, body mass index, current smoking, current drinking, LDL, HDL, triglycerides, kidney disease, ankle/brachial index, interaction of systolic blood pressure, and hypertension treatment.

**Appendix Table 3B:** Association Between Diastolic Blood Pressure SD Quartiles of 8 Nonurgent^a^ Clinic Visits Within a 5-Year Period and Major Adverse Cardiovascular Events

Variable	Unadjusted			Model 1, adjusted^b^			Model 2, adjusted^c^		
	Hazard ratio	95% CI	*P* value	Hazard ratio	95% CI	*P* value	Hazard ratio	95% CI	*P* value
**Diastolic blood pressure SD quartiles (reference, quartile 1)**									
2	0.99	0.77–1.28	.97	1.02	0.82–1.27	.84	0.98	0.78–1.25	.90
3	1.22	0.96–1.57	.11	1.19	0.96–1.47	.11	1.10	0.87–1.39	.45
4	1.24	0.97–1.6	.08	1.18	0.95–1.46	.13	1.11	0.87–1.40	.41
**Strong Heart Study (** 26 **) cohort (reference, Strong Heart Family Study [** 27 **])**									
Strong Heart Study	4.84	3.99–5.86	<.001	1.32	1.03–1.69	.03	1.36	0.97–1.90	.07
**Sex (reference, female)**									
Male	1.69	1.41–2.03	<.001	0.66	0.56–0.77	<.001	0.65	0.54–0.78	<.001
**Strong Heart Study site (reference, Oklahoma)**									
Arizona	0.75	0.53–1.07	.11	0.90	0.68–1.19	.46	0.88	0.62–1.23	.44
South Dakota	2.34	1.87–2.94	<.001	2.13	1.80–2.53	<.001	2.29	1.88–2.79	<.001
**Age at 8th blood pressure, y (continuous)**	1.06	1.05–1.07	<.001	1.06	1.05–1.07	<.001	1.06	1.04–1.07	<.001
**Baseline diastolic blood pressure, mm Hg (continuous)**	1.02	1.01–1.02	<.001	1.01	1.00–1.01	.19	1.01	0.99–1.02	.34
**Hypertension treatment (reference, no)**									
Yes	2.22	1.84–2.67	<.001	NA	NA	NA	1.50	0.42–5.43	.53
**Diabetes (reference, no)**									
Yes	2.64	2.20–3.16	<.001	NA	NA	NA	2.00	1.67–2.40	<.001
**Body mass index (kg/m^2^**) **baseline (continuous)**	0.99	0.99–1.00	.02	NA	NA	NA	1.00	0.98–1.01	.50
**Current smoker (reference, no)**									
Yes	1.30	1.09–1.56	.003	NA	NA	NA	1.41	1.18–1.69	<.001
**Current drinker (reference, no)**									
Yes	0.69	0.58–0.83	<.001	NA	NA	NA	0.85	0.71–1.03	.09
**LDL (reference, <100 mg/dL)**									
100–129 mg/dL	1.18	0.94–1.46	.15	NA	NA	NA	1.05	0.85–1.30	.63
130–159 mg/dL	1.73	1.35–2.22	<.001	NA	NA	NA	1.31	1.03–1.66	.03
160–189 mg/dL	1.60	1.08–2.36	.02	NA	NA	NA	1.01	0.71–1.43	.97
≥190 mg/dL	1.18	0.59–2.36	.63	NA	NA	NA	0.90	0.47–1.74	.76
**HDL (reference, men <40; women <50)**									
Men 40–59 (Women 51–59	0.72	0.58–0.90	.003	NA	NA	NA	1.06	0.85–1.32	.60
Men ≥60 (Women ≥60	0.51	0.38–0.68	<.001	NA	NA	NA	1.00	0.75–1.33	.98
**Triglycerides (reference, <150 mg/dL)**									
150–199	1.31	1.04–1.65	.02	NA	NA	NA	1.09	0.87–1.36	.45
200–499	1.37	1.10–1.72	.005	NA	NA	NA	0.99	0.79–1.23	.91
≥500	1.63	0.91–2.91	.10	NA	NA	NA	1.26	0.73–2.19	.41
**Kidney disease (reference, no)**									
Yes	2.68	2.12–3.39	<.001	NA	NA	NA	1.49	1.19–1.88	<.001
**Ankle/brachial index (reference, >0.9 to ≤1.4)**									
**≤**0.9 or >1.4	2.11	1.51–2.95	<.001	NA	NA	NA	1.34	0.98–1.82	.06

Abbreviations: NA, not applicable; HDL, high-density lipoprotein cholesterol; LDL, low-density lipoprotein cholesterol.

^a^ Excluded hospitalizations, emergency room visits, urgent care visits, ambulance trips, and pregnancy clinical visits.

^b^ Shared frailty Cox proportional hazards model adjusted for cohort, center, age, sex, and systolic blood pressure/diastolic blood pressure as appropriate.

^c^ Shared frailty Cox proportional hazards model adjusted for cohort, sex, center, age, baseline diastolic blood pressure, hypertension treatment, diabetes, body mass index, current smoking, current drinking, LDL, HDL, triglycerides, kidney disease, ankle/brachial index, interaction of systolic blood pressure, and hypertension treatment.
